# Extra Utera Fœtation

**Published:** 1881-10

**Authors:** 


### Domestic Correspondence.

ARTICLE XIII.

MESSRS. EDITORS:—In the August number of the CHICAGO MEDICAL JOURNAL AND EXAMINER, page 178, is published the history of a case of "Extra Uterine Fœtation." As published by Dr. Rozencrans, the case is a very unusual one, and may fairly form the subject of criticism. Permit me to make a few remarks on it.

"Examination of this woman satisfied me that she was a subject for operation; that it would not admit of delay," etc. Yet the "operation" was postponed twenty-three days, and the woman, meantime, brought twenty miles in a wagon. This done, he then, and not till then, makes a thorough examination. "As there was evidently a considerable quantity of fluid in the abdominal cavity," he performed paracentesis abdominis one inch below the navel, drawing off two gallons of dark fluid; he then could feel a "solid substance" in the abdomen. Before making a thorough examination—before tapping—the reporter concluded "she was a subject for an operation," and had the woman removed a distance of twenty miles in a wagon. The data for coming to that conclusion, and for diagnosing an extra-uterine fœtation, are not vouchsafed us, as they might have been. Cases of this kind are obscure enough in their symptoms and signs, and we need further light on the method of diagnosis. Was not the tapping a most dangerous and unwarrantable proceeding? for he tells us, "after cutting through the walls of the abdomen, bringing to view the cyst . . . I . . . at once cut open the cyst," from which flowed the "same character fluid" as had been removed by the trocar. This proves that it was the "cyst" he tapped; that the fluid was not in the abdominal cavity. Was there no peritoneum met "after cutting through the walls of the abdomen," and what was its condition? "I then tore away the placental mass, and proceeded to the removal of the sac." Removed the placenta! Is this the method of dealing with an extra-uterine placenta recommended by Barker, Thomas, Keith, or Spencer Wells? We think not. Where did the placenta grow? Where was the "sac?" and what were its adhesions? What was the condition of the fœtus? He seems to have cut and torn as fearlessly as he would on a cadaver. Why was the right ovary removed?

"To obviate internal bleeding," he, after closing the wound, "placed two bricks on the abdomen for pressure" for half an hour. Were the bricks put on to compress the abdominal aorta? if so, they cannot do it; and to check bleeding in the pelvis they are equally ineffectual. Why close the wound until all bleeding was arrested? From the rough, yes, crude, manner of operating, no one can feel surprise at being told there was "for many days a copious discharge of putrid matter" through the drainage tube, notwithstanding the use of the carbolic spray during the operation. "I applied pounded ice constantly for the space of three weeks to the abdomen." Is it possible for any human being to endure such cold *so long* and live? What was the need of it? How was the peritoneum, of which we hear nothing from beginning to end, dealt with in closing the wound?

Was it absolutely necessary to practice catheterism for a month? Were opiates used? "The temperature of her body remained quite normal," yet she had a pulse of 100 in the morning and of 120 in the afternoon, for about three weeks. What is the explanation of this high pulse-rate? Was the temperature accurately measured two or three times daily by the clinical thermometer? With a "copious discharge of putrid matter" for many days, catheterism for a week, a pulse up to 120 daily for three weeks, and ice to the abdomen constantly for the same time, it does look as though there had been, after all, some peritonitis. Such persistent and prolonged use of ice should be enough to deprive any peritoneum of sensibility. Hence the patient would not complain of pain. The woman was seventy-eight days—a very long time—recovering. What was the cause of this? "I am not aware that a similar operation was ever before performed in this State." We hope not, nor in any other State.

Such is a calm and fair criticism of this case, as reported. What woman, subjected to such conditions, could live after them? When scrutinized, the narrative looks, to me, improbable on the face of it, and beyond the name of Dr. Rozencrans, bears no evidence of authenticity. Is it meant for a rude hoax? Did the operator live in Southern Texas at the time? Did he go from Elgin to Texas to operate? or is he lately come from the part of Texas mentioned to reside in Elgin, Ill.?

---

THE METHOD OF REMOVING FOREIGN BODIES ENGAGED AT THE RIMA GLOTTIDIS.—M. Krishaber (France Med. 16–19, Dec. '80) relates four cases in which the foreign body rested on the superior surface of the vocal cords. In two cases a 50 centime piece was retained by the laryngeal ventricles in a horizontal position. One of these patients was placed upon his abdomen in recumbent posture, the index finger of the left hand passed as far as the edge of the epiglottis, and a pair of laryngeal forceps introduced, but it was impossible to seize the piece, which slipped from the grasp of the instrument. The patient was then placed in front of the operator, a pair of closed laryngeal forceps introduced below the glottic orifice, then opened, being careful to exercise pressure from below, upwards. The piece was dislodged and accidentally swallowed by the patient.

In the second case the piece was pushed out by means of a sound introduced through the tracheal opening made to save life, which was threatened by œdema. A bone was removed from the glottic orifice in the third case by means of forceps.

In the last of the four cases a piece of copper was extracted by the same procedure which failed in the first case mentioned.

Krishaber advises except in the case of infants in whom the larynx is easily reached by the finger of the operator, the performance of tracheotomy, the tamponning of the trachea and the extraction of the foreign body *per vias naturales.*

